# Primary care research on hypertension: A bibliometric analysis using machine-learning

**DOI:** 10.1097/MD.0000000000040482

**Published:** 2024-11-22

**Authors:** Gökben Yasli, Muhammet Damar, Şeyda Özbiçakci, Serkan Alici, Andrew David Pinto

**Affiliations:** a Department of Public Health, İzmir Health Directorate, İzmir, Turkey; b Information Center, Dokuz Eylul University, İzmir, Turkey; c Upstream Lab, MAP, Li Ka Shing Knowledge Institute, Unity Health Toronto, Toronto, Ontario, Canada; d Department of Public Health Nursing, Faculty of Nursing, Dokuz Eylul University, İzmir, Turkey; e Faculty of Economics and Administrative Sciences, Dokuz Eylul University, İzmir, Turkey; f Department of Family and Community Medicine, Faculty of Medicine, University of Toronto, Toronto, Ontario, Canada.

**Keywords:** blood pressure, hypertension, latent Dirichlet allocation, Primary Health Care, topic analyses

## Abstract

Hypertension is one of the most important chronic diseases worldwide. Hypertension is a critical condition encountered frequently in daily life, forming a significant area of service in Primary Health Care (PHC), which healthcare professionals often confront. It serves as a precursor to many critical illnesses and can lead to fatalities if not addressed promptly. Our study underscores the importance of this critical issue by analyzing articles related to hypertension in the PHC research area from the Web of Science Core Collection using bibliometric methods and machine learning techniques, specifically topic analyses using the latent Dirichlet allocation method. The analysis was conducted using Python Scikit-learn, Gensim, and Wordcloud Libraries, the VosViewer program, and the Bibliometrix R Biblioshiny library. Our findings revealed a steady increase in publication output in hypertension-related research. Analysis shows that hypertension-related research in the PHC research area is clustered into 8 groups: (1) management of hypertension in PHC, risk factors, and complications; (2) psychiatric disorders and hypertension; (3) pediatric and pregnancy hypertension; (4) environmental factors and living conditions; (5) sex and age effects on hypertension; (6) COVID-19 and hypertension; (7) behavioral risk factors, quality of life, and awareness; and (8) current treatment methods and guidelines. Research on hypertension has focused intensively on kidney disease, obesity, pregnancy, cardiovascular risk, heart disease, calcium channel blockers, body mass index, amlodipine, mortality, risk factors, hyperlipidemia, depression, and resistant hypertension. This study represents the first and comprehensive bibliometric analysis of hypertension in the PHC research area. Annual publication volumes have steadily increased over the years. In recent years, topics such as social determinants, patient attendance, self-management, diabetes mellitus, COVID-19, telemedicine, type 2 diabetes, and noncommunicable diseases have garnered significant interest in the field of PHC services.

## 1. Introduction

Hypertension is one of the most important chronic diseases worldwide. It is a key risk factor for cardiovascular disease, chronic kidney disease, cognitive decline and is associated with premature death.^[1]^ Hypertension is highly prevalent, affecting one-fourth of the adult male population and one-fifth of the adult female population,^[2]^ with two-thirds of people with hypertension live in low- and middle-income countries.^[3]^ The global economic burden of hypertension is estimated at 10% of total healthcare expenditures worldwide,^[4]^ including direct costs related to hypertension care (e.g., medications, laboratory tests, and clinical visits), and indirect costs due to complications resulting from hypertension and subsequent premature death and disabilities. Hypertension is caused by a combination of genetic factors, high calorie and alcohol intake, smoking, and physical inactivity.^[5]^

Hypertension accounts for 1 out of every 8 consultations in Primary Health Care (PHC).^[6,7]^ Hypertension constitutes a significant risk factor contributing to 60% of premature deaths stemming from noncommunicable diseases.^[8]^ Key barriers to hypertension control include ineffective screening and awareness, difficulties in accessing treatment, challenges in managing hypertension posttreatment, medication adherence barriers, lifestyle-related obstacles, and affordability and accessibility of care.^[9]^ Three out of 4 individuals with hypertension reside in low- and middle-income countries, yet only 10% of those living in such countries have their blood pressure treated and controlled.^[10]^ For instance, only 15% of the hypertensive population in India achieves optimal blood pressure control. Effective monitoring of hypertension is crucial for reducing morbidity and mortality associated with cardiovascular diseases.^[8]^ In 2017, the World Health Organization (WHO) and Resolve to Save Lives designed the WHO HEARTS hypertension service package in 32 low- and middle-income countries, treated 12.2 million patients across 165,000 primary healthcare facilities in HEARTS hypertension control programs by 2022. The experience of this initiative has clearly demonstrated the feasibility and success of controlling hypertension in primary healthcare services in low- and middle-income countries.^[10]^ In other words, this has emphasized the critical importance and effectiveness of primary healthcare in combating hypertension.

To understand the current state of research on the effects of PHC on hypertension, we used bibliometric analyses. Bibliometric analyses refer to the numerical analysis of bibliographic data and may employ correlations, co-citations, and network analyses.^[11]^ Bibliometric analyses can reveal collaboration patterns within a specific field, discover emergent topics of interest, identify prominent subject areas, as well as uncover researchers, authors, institutions, and countries engaged in research.^[12]^ Bibliometric analyses have been used to examine research on specific aspects of hypertension, including arterial hypertension,^[13]^ 100 years of hypertension research,^[14]^ hypertension researches in the west Asia region,^[15]^ exercise for hypertension,^[16]^ hypoxic pulmonary hypertension,^[17]^ pregnancy-induced hypertension,^[18,19]^ acupuncture therapy for hypertension,^[20]^ epigenetics insights of hypertension,^[21]^ trends in worldwide research in hypertension,^[22]^ portal hypertension,^[23]^ and hypertension associated with obstructive sleep apnea.^[24]^ However, no previous work using bibliometric analyses has specifically examined and focused PHC research on hypertension. By revealing complex structures based on large amounts of data, the aim was to provide researchers with a general perspective on hypertension. This enables an in-depth analysis of hypertension, allowing researchers to have a comprehensive understanding of the discussion topics and which topic headings are prominent in the field. Our findings will assist in identifying areas for future PHC research on hypertension.

## 2. Materials and methods

We identified the annual publication outputs, leading countries, articles, journals, institutions, and prominent topic headings in the field of hypertension in PHC through bibliometric analyses. We conducted topic modeling using the latent Dirichlet allocation (LDA) method, allowing for the organization and summarization of high-content textual documents. There is no need to obtain ethical approval. Research data is available to all readers without any restrictions in Web of Science Core Collection.

### 2.1. Data source and research methodology

We used the Web of Science (WoS) Core Collection as the data source. In the last 15 years, WoS, Scopus, and Google Scholar have emerged as the top 3 multidisciplinary bibliographic databases providing metadata on scientific documents and citation links among these documents.^[25]^ However, out of the 3 data sources, only the PHC field as “Primary Health Care” is directly associated on WoS. Thomson Reuters WoS announced the “Primary Health Care” Subject Category for the first time in 2011, and in that year, 13 journals were indexed within this category. This subject category enables a better elucidation of scientific production in primary healthcare services and family medicine.^[26]^ As of today, it has been observed that 33 journals in the relevant field are indexed by WoS.

**Figure 1.**
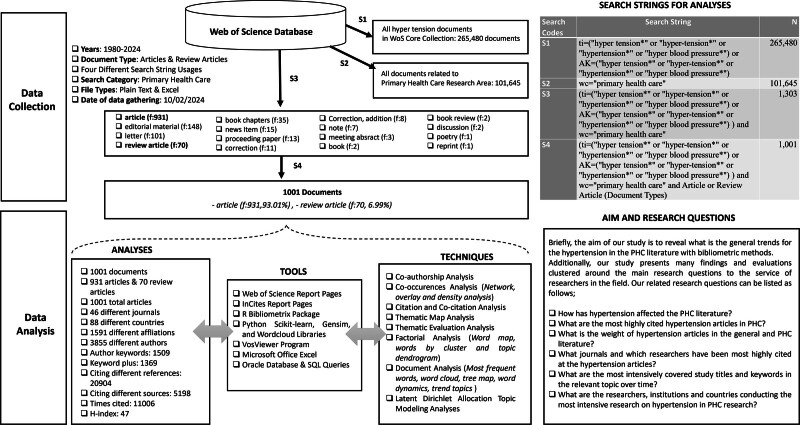
Research methodology and study design.

On WoS, there are 265,480 documents where the term “hypertension” appears either in the title or as a researcher keyword. Among these documents, 1303 were related to the research field of Primary Health Care (PHC), with 1001 of them being articles. In the PHC research field, there were 101,645 documents. Thus, 1 in every hundred documents in the PHC research field is associated with hypertension. We used data obtained from the WoS bibliometric data source and included all articles within PHC research containing the word “hypertension” in the article title or researcher keywords. We used the following tools for our analysis: MS Excel, Oracle Database, R Bibliometrix Biblioshiny, and VosViewer. Additionally, WoS and Incite reporting tools have been utilized at various points. The data were collected on February 10, 2024, following the filtering process indicated in Table 1 in Figure 1, and the dataset relevant to query number 4 was extracted in the final stage.

**Table 1:** Cluster title and description of LDA topic modeling findings for abstracts and titles.

Cluster title	Narrative description of content
Management, risk factors, and complications of hypertension in PHC	The words in this cluster define the risk factors, complications, and management of hypertension treatment.
Psychiatric disorders and hypertension	Psychological factors such as anxiety and depression have been discussed under this heading in relation to hypertension and PHC.
Pediatric and pregnancy hypertension	Under this heading, research related to the definition of hypertension, risk factors, diagnostic methods, the effect of medications used during pregnancy on hypertension, and referral situations in children and pregnant women has been grouped.
Environmental factors and living conditions	In this cluster, the impact and significance of a person’s hypertension on their work life, social life, environment, and other living conditions in PHC are described.
The effect of gender and age on hypertension	Research on the prevalence of hypertension based on gender and age groups is clustered under this heading.
COVID-19 and hypertension	Studies investigating the interaction between hypertension prevalence and diseases such as diabetes, kidney failure, in patients recovering from COVID-19 pneumonia are categorized under this heading.
Behavioral risk factors, quality of life, and awareness	Research examining the relationship between behavioral risk factors for hypertension and awareness is categorized under this heading.
Current treatment methods and guideline resources	Approaches to hypertension treatment, health management, types of medications, guidelines developed in the field of PHC, and hypertension control are grouped under this heading.

LDA= latent Dirichlet allocation, PHC= Primary Health Care.

### 2.2. LDA for topic modeling

Unsupervised machine learning trains computers to uncover hidden structures and identify specific patterns within unlabeled data without predetermined variables.^[27,28]^ LDA is an unsupervised learning algorithm that does not require predefined terms. Once the number of topics is determined, the topics are labeled according to the class. LDA is a probability-based topic modeling method that generates topics based on word weights from a set of documents.

Topic modeling is used to define relationships between text data.^[29]^ Analyzing textual data using unsupervised machine learning can be understood as the principal component analysis of social sciences and is actively applied in the healthcare field to determine the underlying meaning of textual data.^[28]^ For instance, it has been applied in various areas such as the impact of the COVID-19 pandemic on research in nursing,^[30]^ sentiment prediction for diabetes mobile applications,^[31]^ personal goals among patients undergoing bladder cancer surgery,^[32]^ insights into Korean public perspectives on urology,^[33]^ modeling research topics for artificial intelligence applications in medicine,^[34]^ cardiology record analyses,^[35]^ identifying latent topics and trends in premature infant-related nursing studies,^[36]^ and predicting clinical trial terminations.^[37]^ Similar to these studies, extracting main themes or topics from large document collections, identifying popular and emerging topics, and using text analysis are common practices.^[38]^ In other words, these models can transform information from a collection of thousands of documents into a focused summary.^[39]^

## 3. Results

### 3.1. General statistics

**Figure 2.**
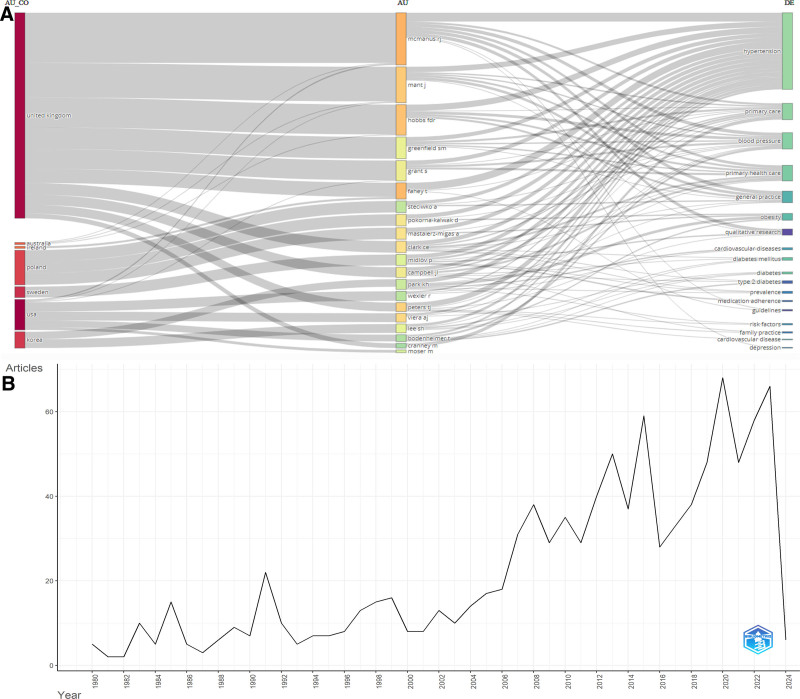
Three field of author country, author, and author keywords (A), annual scientific production (B) for hypertension articles in PHC research area. PHC= Primary Health Care.

From 1980 to the present, 1001 articles related to hypertension in the field of PHC research have been published, consisting of 931 research articles and 70 review articles. The indexes where the relevant documents were scanned were as follows: Science Citation Index Expanded, 696 articles; Emerging Sources Citation Index, 274 articles; and Social Sciences Citation Index, 86 articles. The total number of citations received by the documents was 11,006 (without self-citations: 10,624), with an average citation count of 11 and h-index (HI) value of 47. Overall, interest in hypertension has increased over the years (see Fig. 2B).

When the publications on the subject were sorted by the languages in which they were published, 897 articles (89.61%) were in English. Following English, Polish (42 articles, 4.19%), Spanish (40 articles, 3.99%), Korean (16 articles, 1.59%), and French (6 articles, 0.59%) were ranked. Additionally, 54.74% (548 articles) of the studies on hypertension were published as open-access.

### 3.2. Country, institution, and author analysis

When examining the studies, it was observed that 1001 articles were produced by 3855 different researchers from 1591 different institutions in 88 different countries. The top 5 countries with the most intense research activity in the field were the United States of America (USA) (document count [N]: 327, 32.66%; average citations per article [ACPA]: 11.92), England (N: 110, 10.98%, ACPA: 23.24), Canada (N: 76, 7.59%, ACPA: 13.22), India (N: 73, 7.29%, ACPA: 3.00), and Spain (N: 57, 5.69%, ACPA: 6.91) (Appendix 1, Supplemental Digital Content, http://links.lww.com/MD/N879). The top 5 countries with the most citations were the USA, England, Canada, Scotland, and Sweden. In the field of PHC research, the top 5 institutions engaged in hypertension research were the University of Oxford (England, N: 25), University of California System (USA, N: 22), University of Toronto (Canada, N: 22), University System of Ohio (USA, N: 22), and Lund University (Sweden, N: 4620) (Appendix 2, Supplemental Digital Content, http://links.lww.com/MD/N879). The value of international co-authorship was 9.89%, that of single-authored documents was 121, and coauthors per document was 4.53. The relevant researchers, their countries, and the keywords frequently used in their articles are shown in Figure 2A.

**Figure 3.**
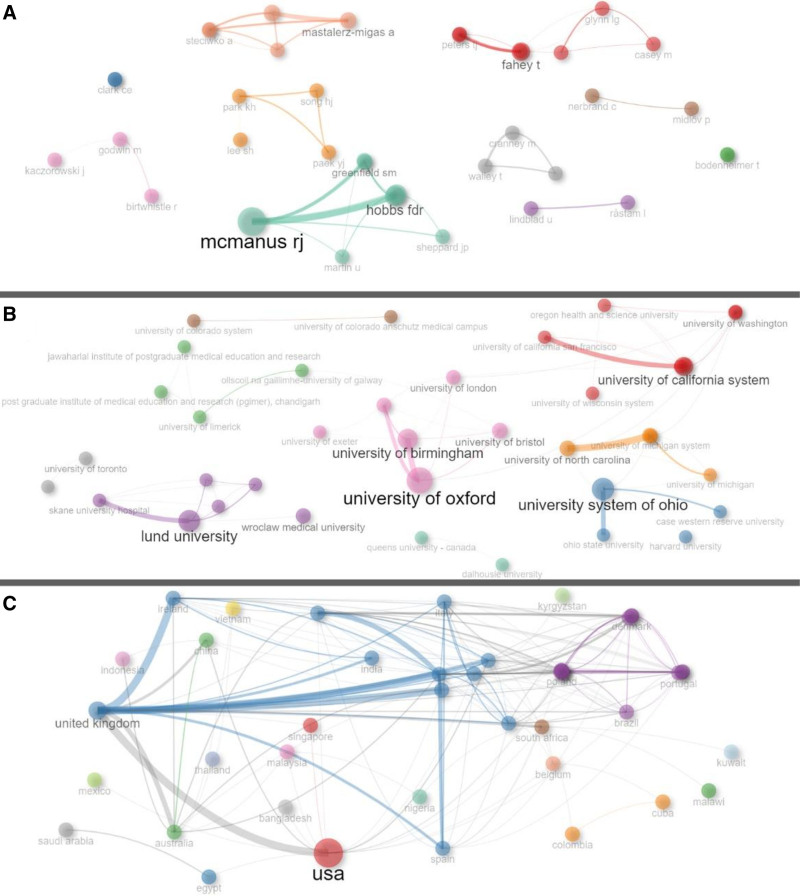
Author (A), affiliations (B) and country (c) collaborations for hypertension articles in PHC research area. PHC= Primary Health Care.

The top 9 most prolific researchers in the relevant field and topic are as follows: Mcmanus RJ (University of Oxford, England, ACPA: 13.00, N: 15), Fahey T (Royal College of Surgeons, Ireland, ACPA: 47.73, N: 11), Hobbs FDR (University of Oxford, England, ACPA: 14.00, N: 9), Mant J (University of Cambridge, England, ACPA: 14.78, N: 9), Peters TJ (University of Bristol, England, ACPA: 35.75, N: 8), Viera AJ (Duke University, USA, ACPA: 27.88, N: 8), Clark CE (University of Exeter, England, ACPA: 30.14, N: 7), Mastalerz-migas A (Wroclaw Medical University, Poland, ACPA: 0.86, N: 7), Pokorna-kalwak D (Wroclaw Medical University, ACPA: 0.86, N: 7). Figure 3 shows the collaboration among researchers (Fig. 3A), institutions (Fig. 3B), and countries (Fig 3C).

### 3.3. Document and reference analysis

The 1001 documents were published in 46 different journals, receiving 11,006 citations, with an average citation count of 11.00 and an HI value of 47. Among the prominent journals in the field, where articles on hypertension in the PHC research area are most frequently published, the top 5 journals are as follows: British Journal of General Practice (Journal Impact Factor for 2022 [JIF]: 5.90), Journal of Family Medicine and Primary Care (JIF: 1.40), Family Practice (JIF: 2.20), BMC Family Practice (JIF: 2.90), American Family Physician (JIF: 4.00) (Appendix 3, Supplemental Digital Content, http://links.lww.com/MD/N879). Among the top 20 journals where studies on hypertension in the PHC research area were most frequently published, it was observed that only 6 journals were indexed in the Emerging Sources Citation Index, while the other 14 journals were indexed in the Science Citation Index Expanded.

Upon examining the top 15 most-cited articles in the PHC research area and hypertension field, it was found that 5 articles were published in the British Journal of General Practice and Annals of Family Medicine, 2 in Family Practice, and 1 each in the Journal of Family Practice, American Family Physician, and BMC Family Practice (Appendix 4, Supplemental Digital Content, http://links.lww.com/MD/N879). Additionally, among the top 10 journals with the most citations in articles on PHC and hypertension, the following were observed: hypertension (Citation [C]: 1153), Lancet (C: 959), Journal of Hypertension (C: 927), Journal of the American Medical Association (C: 920), The New England Journal of Medicine (C: 720), Circulation (C: 631), British Medical Journal (C: 541), Archives of Internal Medicine (C: 528), American Journal of Hypertension (C: 455), and Annals of Internal Medicine (C: 399).

According to Bradford Law,^[40,41]^ the journals in the first zone are the British Journal of General Practice, Journal of Family Medicine and Primary Care, Family Practice, BMC Family Practice, and American Family Physicians. Following this, the journals in the second zone are Primary Care, Family Medicine and Primary Care Review, Canadian Family Physician, Journal of Family Practice, Journal of The American Board of Family Medicine, Scandinavian Journal of Primary Health Care, and Korean Journal of Family Medicine. In particular, journals in the first zone can be considered core journals that researchers in the field of PHC services research on hypertension should primarily follow. Furthermore, the increase in the number of publications on the relevant topic over the years in these journals, particularly in the first zone, serves as additional evidence to support this assertion.

### 3.4. Keywords, titles and abstracts analyses, and emerging trends

In the 1001 articles on hypertension conducted in the field of PHC services research, 1001 abstracts and titles, 1509 author keywords, and 1369 keywords plus keywords were used. Additionally, these studies were associated with 17 research areas outside the PHC. The related research areas, in order of frequency, are as follows: medicine general internal (N: 639, 63.83%), health care sciences services (N: 47, 4.69%), endocrinology metabolism (N: 23, 2.29%), orthopedics (N: 23, 2.29%), sport sciences (N: 23, 2.29%), cardiac cardiovascular systems (N: 14, 1.39%), public environmental occupational health (N: 6, 0.59%), health policy services (N: 4, 0.40%), nutrition dietetics (N: 2, 0.20%), urology nephrology (N: 2, 0.20%), emergency medicine (N: 1, 0.10%), ethnic studies (N: 1, 0.10%), genetics heredity (N: 1, 0.10%), medicine research experimental (N: 1, 0.10%), obstetrics gynecology (N: 1, 0.10%), pediatrics (N: 1, 0.10%), physiology (N: 1, 0.10%).

**Figure 4.**
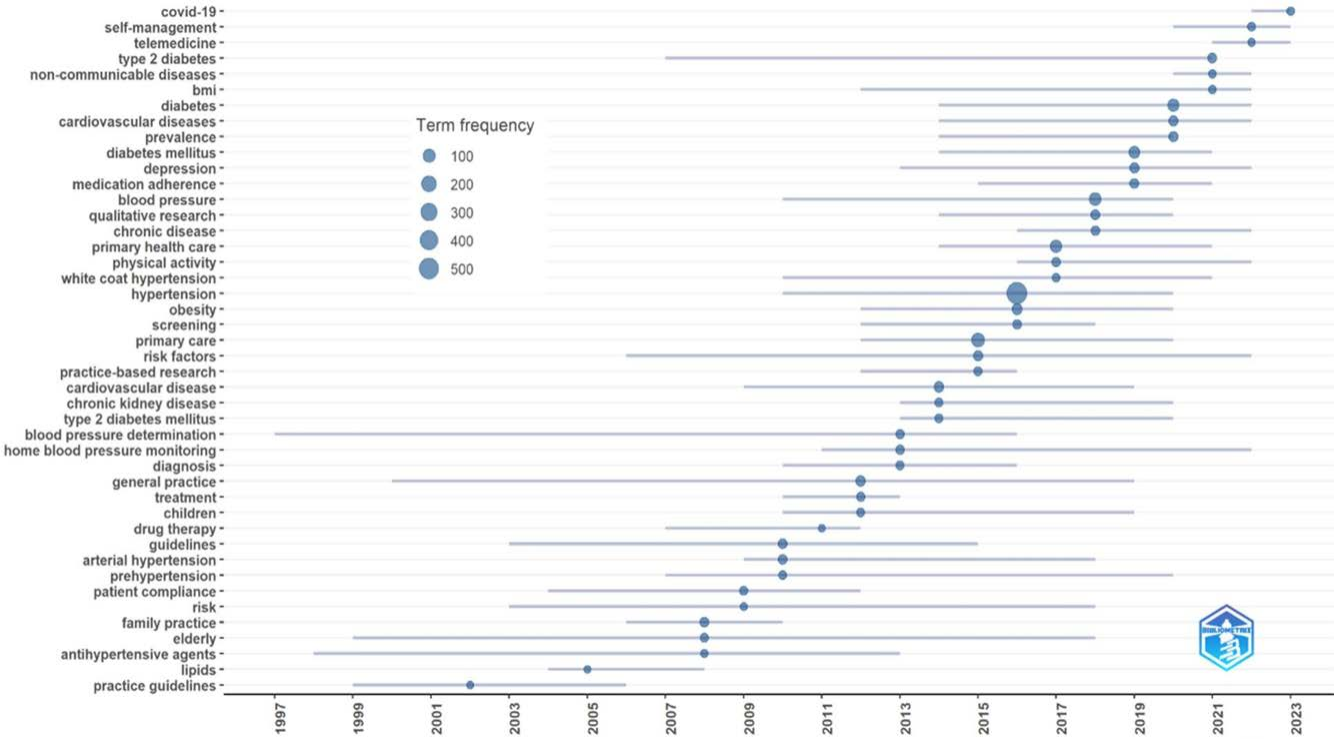
Trend topic analyses based author’s keyword.

Over the years, prominent discussion topics on hypertension in the field of PHC services research have been presented based on author keywords in Figure 4. The keywords, headings and abstracts in an article are carefully prepared by the authors of the article in a way that most clearly expresses the characteristics of the study. Keywords, title, and abstract of articles provided us with the opportunity for a more detailed analysis of the filtered articles in the relevant field. Analyses conducted on the words used can provide us with a wealth of information about current research areas, heavily discussed topics, the types of topics that have been heavily addressed at different times, and the relationships between different topics, depending on the analyses conducted. In our study, analyses conducted in this direction revealed that in recent years, in the field of PHC services, topics such as COVID-19, self-management, prevention, blood pressure, prevalence, cardiovascular disease, risk factors, telemedicine, type 2 diabetes, obesity, physical activity, therapy, quality of life, noncommunicable diseases, antihypertensive drugs, losartan, target organ damage, and guidelines have received significant attention. Furthermore, in our study, researchers in the field of PHC services focused on topics such as kidney disease, obesity, pregnancy, cardiovascular risk, heart disease, calcium channel blockers, body mass index, amlodipine, mortality, risk factors, obstructive sleep apnea, hyperlipidemia, depression, anxiety, and resistant hypertension in the context of hypertension.

### 3.5. LDA analysis results

Eight different topics emerged (Table 1, Appendix 5, Supplemental Digital Content, http://links.lww.com/MD/N879): (1) management, risk factors, and complications of hypertension in PHC; (2) psychiatric disorders and hypertension; (3) pediatric and pregnancy hypertension; (4) environmental factors and living conditions; (5) the effect of sex and age on hypertension; (6) COVID-19 and hypertension; (7) behavioral risk factors, quality of life, and awareness; and (8) current treatment methods and guideline resources.

## 4. Discussion

Despite numerous bibliometric analyses conducted on hypertension in the literature, there has been a lack of critical analysis of hypertension, which holds significant value in the field of PHC services. In this study, a map of the productivity trends of hypertension articles in the PHC research field was generated. It has been revealed that hypertension is a heavily researched topic in the relevant field. The total number of citations received by the documents and the high HI value of 47 supports this observation. As emphasized by Mao et al, fluctuations in the quantity of academic articles in a specific research field serve as important indicators of the field’s development.^[42]^ Plotting the graph of publication quantity over time and conducting statistical analyses (i.e., trend topics analyses for titles, abstracts, and author keywords over the years) contribute to understanding the research level of research and future trends. Hirsch developed the HI and defined a successful scientist with an HI of 20 as a successful scientist and 40 as an outstanding scientist.^[43]^ This definition can be extended to research fields. Researchers in career-themed research in nursing have a high HI value of 47.^[12]^ Therefore, according to the approach of Hirsch and Bilik et al, the topic of hypertension in the field of PHC is a highly regarded subject in research literature.^[9,43]^ With the increasing life expectancy worldwide, hypertension will continue to be a significant health issue in PHC. The increase in the number of publications on the relevant topic over the years further confirmed this observation.

In the USA, 1 out of every 2 individuals aged 20 and above has hypertension, and only 39.64% of those using medications for hypertension have it well controlled.^[44]^ Hypertension and upper respiratory tract infections are the most common reasons for PHC visits in the USA.^[45]^ The significant public health issue of hypertension in the USA is reflected in scientific productivity. The USA and England are the most active countries in conducting research and receiving citations in the relevant fields. This situation may be associated with financial support for scientific research in developed countries.^[46,47]^ Publications with addresses in the USA also receive a considerable number of citations, likely because of the leading position of US universities in international ranking systems and the presence of institutions that attract expert researchers in the field.^[48,49]^ This phenomenon is supported by the researcher, institution, and country networks shown in Figure 3. Clustering around specific countries, institutions, and researchers is shown in the figure. In terms of scientific output, researchers from the University of Oxford and Royal College of Surgeons have been the most productive in relevant fields and topics. Researchers aiming to increase their knowledge in the field may focus on productive authors and heavily cited studies. In addition, field researchers can establish connections with relevant institutions and researchers to advance their research in the field during and after their doctoral studies.

When examining trends in research related to hypertension, it was observed that interest in the topic has generally increased over the years. The impact factor of journals is an important reference value for researchers to receive more citations of their work. This trend has been repeated in hypertension research, and the most cited articles are typically published in journals with high impact factors. Additionally, the British Journal of General Practice and the Journal of Family Medicine and Primary Care are the 2 most prominent journals in the field according to Bradford law. According to Bradford law,^[40,41]^ these journals can be considered priority journals for researchers interested in hypertension in the PHC research field.

Although hypertension can be easily diagnosed and treated with simple and low-cost medications, there are significant gaps between diagnosis and treatment. Developments in the detection, treatment, and control of hypertension have varied considerably between countries. Some middle-income countries now outperform high-income countries. Sustainable, community-based strategies and programs are needed for a country to control hypertension.^[50]^ National representative data should be collected at regular intervals to better understand differences in control rates between regions and subpopulations within a country.^[51]^ Bhagani et al have emphasized the necessity of implementing national health policies to address this issue, stating that well-organized programs, guidelines, resources, and policy development will lead to improvements in the prevention, detection, and treatment of hypertension.^[7]^ Our study as also as similar findings. Topics such as guidelines, disease management, self-management, and education as well as guideline development,^[52]^ European guidelines,^[53,54]^ practice guidelines and hypertension guidelines,^[55]^ PHC guidelines,^[56]^ education program guidelines,^[57]^ clinical-practice guidelines,^[58,59]^ hypertension programs,^[60,61]^ and self-management,^[62–64]^ have been extensively covered by researchers working in the field.

As revealed by LDA analysis, hypertension has been studied by mental health researchers for years owing to its etiology involving emotional factors and the emergence of psychiatric symptoms during its course. The exact cause of essential hypertension remains unknown. Chronic stress is considered a risk factor for hypertension, with anger, anxiety, depression, and stress being the other contributing factors. The intensity and duration of exposure to chronic stress are significant risk.^[65]^ Additionally, anger, excessive alcohol consumption, smoking, and high body mass index values are associated with adverse lifestyle behaviors and are considered critical behavioral risk factors for hypertension and cardiovascular diseases.^[66]^

Pediatric hypertension is an important and increasingly prevalent health issue.^[67,68]^ While it is rare in the pediatric population, recent studies have shown a significant increase in its frequency, largely due to the obesity epidemic. Our research indicates that pediatric hypertension and obesity are heavily addressed topics in hypertension-related studies conducted in PHC settings. A large cohort study involving over 100,000 children and adolescents followed for several years revealed that those with obesity and severe obesity initially had higher blood pressure levels and were more likely to develop hypertension in later years compared to those in lower body mass index categories.^[69]^ Individuals with pediatric hypertension extending into adulthood are likely to carry cardiovascular and kidney diseases, making early diagnosis and treatment crucial due to its status as a risk factor. Therefore, health care providers working in PHC settings and following such patients should be vigilant. Moreover, when pharmacological treatment is necessary, drug selection should be based on careful evaluation of side-effect profiles and etiology.^[70–73]^ Additionally, our analysis highlights hypertension during pregnancy as another intensively addressed topic. It is well-known that the risk of hypertension recurrence in subsequent pregnancies is approximately 70% for women with a history of pregnancies complicated by gestational hypertension, necessitating regular doctor visits for long-term cardiovascular risk monitoring.^[74]^ Therefore, blood pressure measurement and monitoring during prenatal care in PHC settings are also important in this regard.

It has been observed that articles on hypertension have been produced by 3855 different researchers from 1591 different institutions across 88 different countries. Evidently, the topic receives attention from various countries, institutions, and researchers in the field of PHC research. This can be attributed to the fact that hypertension is a global public health issue. In their study, Zhou et al stated that more than 1 billion hypertensive individuals reside in low- and middle-income countries and that treatment and control rates have improved in most countries since 1990.^[50]^ They noted that improvements were most pronounced in high-income countries, followed by Central Europe, Costa Rica, Taiwan, Kazakhstan, South Africa, Brazil, Chile, Turkey, and Iran, among other countries and regions. Epidemiological studies have consistently demonstrated a graded relationship between socioeconomic status and the risk of hypertension, cardiovascular disease, and mortality. In a study conducted in 2 geographically distinct regions with vastly different socioeconomic conditions (urban Singapore and rural India), the prevalence of hypertension, awareness, and treatment was higher among Indians living in Singapore than in those living in rural areas of India. Overall, these studies indicate a decrease in the awareness, treatment, and control of hypertension in poor living conditions.^[75]^ Socioeconomic factors account for some of the observed differences between the 2 communities. Saif-Ur-Rahman et al confirmed the effectiveness of non-pharmacological interventions in the prevention of hypertension in studies conducted in low- and middle-income countries.^[76]^ This information is valuable for developing public health strategies.

The issue of aging is becoming increasingly serious and persists throughout the century. High blood pressure or hypertension is a prevalent health problem among older adults, and our research highlighted hypertension as a prominent topic in old age. Dai et al stated that the prevalence of hypertension increases linearly with age.^[77]^ As the number of older adults with hypertension is expected to rise, elderly individuals with hypertension should be the focus of health policies. Wu et al found that the relationship between stage 3 hypertension and mortality is stronger in elderly individuals aged 60 to 69.^[78]^ The management of hypertension requires comprehensive assessment, including established coronary artery disease, atrial fibrillation, and stroke patients. In this evaluation, patient preferences, medical comorbidities, life expectancy, treatment goals, and shared decision making between clinicians and patients were critical factors.

Sex differences observed in hypertension stem from both biological and behavioral factors. Our research has shown that sex differences contribute to the development of hypertension. Among biological factors, protective sex hormones in women, chromosomal differences, and other biological sex disparities play a role, whereas behavioral risk factors include high body mass index, smoking, and low physical activity. When considered together, behavioral differences may account for sex disparities in hypertension. However, behavioral differences such as smoking exacerbate inequality.^[79]^ As demonstrated in combined analyses of various cohort studies, and gender disparity is evident in various cardiovascular diseases, including myocardial infarction, heart failure, and stroke. These findings raise the question of whether gender-specific thresholds for hypertension definition are necessary to address the increased risk associated with high blood pressure in women.^[80]^ Therefore, these findings underscore the critical importance of regular medical visits to increase hypertension awareness and reduce gender disparities in cardiovascular health.

Zhou et al reported that the most common comorbidities accompanying COVID-19 were hypertension (30%), diabetes (19%), and coronary heart disease (8%).^[81]^ Additionally, there is no evidence suggesting that the use of ACE inhibitors or ARBs is harmful or beneficial during the COVID-19 pandemic. These agents should be used to control the blood pressure. At least for now, based on the current evidence, they should not be discontinued. Furthermore, a significant number of people with comorbidities such as diabetes and hypertension have experienced considerable challenges during the COVID-19 pandemic.^[82]^

The emergence of hypertension and other cardiovascular diseases is strongly associated with various risk factors, such as aging populations, family history, socioeconomic changes supporting sedentary lifestyles, obesity, smoking, alcohol consumption, unhealthy dietary habits, and stress. The WHO has indicated that the primary behavioral risk factors for noncommunicable diseases are physical inactivity, unhealthy eating, tobacco use, and alcohol consumption. These risk factors can be prevented.^[83]^ Discrepancies in blood pressure measurements have been observed in research studies, sometimes due to medical reasons and sometimes due to lifestyle factors.^[84,85]^ Topics such as body mass index, physical activity, obesity, and sedentary lifestyle have been intensively discussed in relation to hypertension.^[86–89]^ PHC professionals should identify patients with high normal or unstable blood pressure in clinical practice, assess them for overall hypertension risk, and provide personalized lifestyle change recommendations for the early prevention of significant hypertension and cardiovascular disease. Patients who are well-informed about their condition are more motivated to monitor their blood pressure. Identifying individuals in need of preventive measures to reduce the risk of developing hypertension is easier within the PHC system. In this regard, PHC providers play an important role.

One of the most important strategies in hypertension management is to provide care services with a multidisciplinary team (including personal care and monitoring, basic care practices, lifestyle recommendations and counseling, and systematic monitoring). Generally, patients are not solely responsible for low control rates of hypertension. Since hypertension often manifests without symptoms, healthcare providers in a busy clinical setting may easily overlook it.^[89,90]^ Lindblad et al stated in their study that 3 consecutive measurements are sufficient for diagnosing hypertension.^[91]^ However, it has been observed that blood pressure can vary, especially between different clinic visits, as evidenced in the relevant literature. Therefore, it is important to conduct blood pressure monitoring with measurements taken under the same conditions at the same center. The importance of PHC services and guidelines has been reiterated. Countries that base their health systems on PHC tend to have better public health outcomes, lower health inequalities, more equitable access to care and lower costs. Hypertension can be easily detected through blood pressure measurements at home or in healthcare centers and can often be effectively treated with low-cost medications.

In our research, antihypertensive drugs, losartan, amlodipine, and microalbuminuria were observed to be another intensively discussed topic in hypertension.^[92,93]^ The medications for hypertension have been discussed in various contexts, such as their interaction with pregnancy-induced hypertension,^[94]^ the interaction between diabetes and hypertension,^[93,95,96]^ and the development of selected cardiovascular diseases with the use of nonsteroidal anti-inflammatory drugs.^[97]^

## 5. Strength and limitation

This study represents the first bibliometric analysis conducted on hypertension in the PHC research field as well as the first bibliometric analysis conducted using machine learning techniques in the PHC field. In our research, we identified the current status of the field, created visual maps, described the overall situation of the field in 8 different topics using LDA topic modeling analysis, and examined trends and current topics to provide references for future research. However, our study had some limitations. First, all data were obtained from the WoS Core Collection. Although WoS indexes the most important journals in the field, there may be journals that are not. Second, only English-published studies were included in the integrity analysis. Third, our research only analyzed articles and review article types. The primary reason is that these document types are the most suitable for providing the essence of the field, and they are the richest bibliometric data types in terms of metrics such as abstracts, titles, keywords, and references.

## 6. Conclusion

In this study, comprehensive bibliometric and topic analyses were conducted to visualize and evaluate the temporal dynamics and research trends of hypertension-related research in the PHC services research domain using the WoS bibliometric database. Our study represents the most comprehensive research on hypertension within the focus of PHC services. We focused on discussions related to hypertension in PHC services and evaluated current and interesting research in the field. Additionally, this study serves as an important resource for topic modeling of hypertension to guide researchers and practitioners in their future studies and provide further insights into the subject.

Hypertension is a prominent topic in the literature, which holds true for the field of PHC service research as well. Annual publication volumes have steadily increased over the years. According to Bradford law, the British Journal of General Practice and the Journal of Family Medicine and Primary Care are the top 2 journals in the field. In the analysis conducted using LDA topic modeling, it was observed that research in the field of PHC concentrated on 8 different topics. In recent years, topics such as social determinants, patient attendance, self-management, diabetes mellitus, COVID-19, telemedicine, type 2 diabetes, and noncommunicable diseases have garnered significant interest in the field of PHC services. Researchers in the field of PHC services have focused their studies on topics such as kidney disease, obesity, pregnancy, cardiovascular risk, heart disease, calcium channel blockers, body mass index, amlodipine, mortality, risk factors, hyperlipidemia, depression, and resistant hypertension in relation to hypertension.

## Acknowledgments

M. Damar was supported by the Scientific and Technological Research Council of Türkiye (TUBITAK) under the TUBITAK 2219 International Postdoctoral Research Fellowship program. He would like to thank the Upstream Lab, MAP, Li Ka Shing Knowledge Institute at the University of Toronto for its excellent hospitality.

## Author contributions

**Conceptualization:** Gökben Yasli, Muhammet Damar, Serkan Alici, Andrew David Pinto.

**Data curation:** Gökben Yasli, Muhammet Damar, Şeyda Özbiçakci, Serkan Alici, Andrew David Pinto.

**Formal analysis:** Gökben Yasli, Muhammet Damar, Şeyda Özbiçakci, Serkan Alici, Andrew David Pinto.

**Funding acquisition:** Muhammet Damar, Şeyda Özbiçakci.

**Investigation:** Muhammet Damar, Şeyda Özbiçakci, Serkan Alici, Andrew David Pinto.

**Methodology:** Muhammet Damar, Şeyda Özbiçakci, Serkan Alici, Andrew David Pinto.

**Project administration:** Muhammet Damar.

**Resources:** Gökben Yasli, Muhammet Damar, Şeyda Özbiçakci, Serkan Alici, Andrew David Pinto.

**Software:** Muhammet Damar, Serkan Alici, Andrew David Pinto.

**Supervision:** Muhammet Damar, Andrew David Pinto.

**Validation:** Muhammet Damar, Şeyda Özbiçakci, Andrew David Pinto.

**Visualization:** Muhammet Damar.

**Writing – original draft:** Gökben Yasli, Muhammet Damar, Şeyda Özbiçakci, Andrew David Pinto.

**Writing – review & editing:** Gökben Yasli, Muhammet Damar, Şeyda Özbiçakci, Andrew David Pinto.

## Supplementary Material


