# Supplementary material for: Primary care research on hypertension: A bibliometric analysis using machine-learning
Source: Medicine (Baltimore). 2024 Nov 22;103(47):e40482. doi: 10.1097/MD.0000000000040482 (PMC11596423; doi:10.1097/MD.0000000000040482)

Appendix 1. Top 30 Most Intensive Countries on Hypertension in the PHC Research Area

Rank	Countries	HI	ACPA	N	%	Rank	Countries	HI	ACPA	N	%
1	USA	32	11.92	327	32.66	16	Malaysia	7	11.20	10	0.99
2	England	29	23.24	110	10.98	17	Saudi Arabia	3	2.50	10	0.99
3	Canada	17	13.22	76	7.59	18	Switzerland	6	13.40	10	0.99
4	India	8	3.00	73	7.29	19	Finland	6	8.44	9	0.89
5	Spain	9	6.91	57	5.69	20	Iran	2	1.67	9	0.89
6	Poland	5	1.28	53	5.29	21	Ireland	6	17.00	9	0.89
7	South Korea	10	8.74	41	4.09	22	Denmark	5	10.63	8	0.79
8	Sweden	12	12.00	39	3.89	23	Israel	6	16.50	8	0.79
9	South Africa	10	8.74	34	3.39	24	Belgium	4	16.29	7	0.69
10	Australia	12	15.19	26	2.59	25	France	4	18.71	7	0.69
11	Netherlands	14	16.15	26	2.59	26	Nigeria	4	9.14	7	0.69
12	China	10	10.50	24	2.39	27	Brazil	4	5.33	6	0.59
13	Scotland	14	30.68	19	1.89	28	Hungary	4	10.83	6	0.59
14	Germany	9	17.29	17	1.69	29	Italy	6	12.83	6	0.59
15	Norway	9	19.23	13	1.29	30	Turkey	5	10.00	6	0.59

ACPA: Average citation per articles, N: Document count, HI: H-index.

Appendix 2. Top 15 Institutions Working Most Intensively on Hypertension in the PHC Research Area

Rank	Affiliations	Country	TC	HI	ACPA	N	%
1	University of Oxford	England	426	12	17.04	25	2.49
2	University of California System	USA	356	8	16.18	22	2.19
3	University of Toronto	Canada	1,005	17	13.22	22	2.19
4	University System of Ohio	USA	147	8	6.68	22	2.19
5	Lund University	Sweden	284	9	14.20	20	1.99
6	Wroclaw Medical University	Poland	33	3	1.74	19	1.89
7	University of Birmingham	England	268	9	14.89	18	1.79
8	University of Bristol	England	612	13	34.00	18	1.79
9	University of London	England	172	7	12.29	14	1.39
10	University of North Carolina	USA	258	8	21.50	12	1.19
11	Skane University Hospital	Sweden	156	8	14.18	11	1.09
12	University of Gothenburg	Sweden	81	5	7.36	11	1.09
13	University of Washington	USA	43	4	3.91	11	1.09
14	Ohio State University	USA	86	5	8.60	10	0.99
15	University of California San Francisco	USA	290	6	29.00	10	0.99
16	University of Cambridge	England	165	8	16.50	10	0.99
17	University of Exeter	England	311	7	31.10	10	0.99
18	University of Washington Seattle	USA	40	4	4.00	10	0.99
19	US Department of Veterans Affairs	USA	106	5	10.60	10	0.99
20	Yale University	USA	52	5	5.20	10	0.99

ACPA: Average citation per article, N: Document count, HI: H-index, TC: Times cited.

Appendix 3. The 20 Journals with the Highest Number of Articles on Hypertension in the PHC Research Area

Rank	Journal	JIF	Research Domain	SCIE/ SSCI/ ESCI	ACPA	HI	N	%
1	British Journal of General Practice	5.90	Medicine, General & Internal; Primary Health Care	SCIE & SSCI	23.85	26	86	8.59
2	Journal of Family Medicine And Primary Care	1.40	Primary Health Care	ESCI	2.62	8	84	8.39
3	Family Practice	2.20	Medicine, General & Internal; Primary Health Care	SCIE & SSCI	19.23	20	69	6.89
4	BMC Family Practice	2.90	Medicine, General & Internal; Primary Health Care	SCIE & SSCI	18.61	20	61	6.09
5	American Family Physician	4.00	Medicine, General & Internal; Primary Health Care	SCIE & SSCI	14.47	17	59	5.89
6	Primary Care	1.90	Medicine, General & Internal; Primary Health Care	SCIE & SSCI	6.86	11	59	5.89
7	Family Medicine And Primary Care Review	0.70	Primary Health Care	ESCI	0.86	4	58	5.79
8	Canadian Family Physician	3.10	Medicine, General & Internal; Primary Health Care	SCIE & SSCI	6.67	10	57	5.69
9	Journal of Family Practice	0.60	Medicine, General & Internal; Primary Health Care	SCIE & SSCI	10.63	12	49	4.89
10	Journal of The American Board of Family Medicine	2.90	Medicine, General & Internal; Primary Health Care	SCIE & SSCI	13.98	14	43	4.29
11	Scandinavian Journal of Primary Health Care	2.10	Health Care Sciences & Services; Medicine, General & Internal; Primary Health Care	SCIE & SSCI	13.74	14	43	4.29
12	Korean Journal of Family Medicine	2.30	Primary Health Care	ESCI	6.78	9	40	3.99
13	Atencion Primaria	2.50	Medicine, General & Internal; Primary Health Care	SCIE & SSCI	5.34	8	38	3.79
14	African Journal of Primary Health Care Family Medicine	2.00	Primary Health Care	ESCI	7.71	10	34	3.39
15	Journal of Primary Care And Community Health	3.60	Primary Health Care	ESCI	5.18	8	33	3.29
16	Annals of Family Medicine	4.40	Medicine, General & Internal; Primary Health Care	SCIE & SSCI	48.85	19	27	2.69
17	BMC Primary Care	-	Medicine, General & Internal; Primary Health Care	SCIE & SSCI	1.12	3	26	2.59
18	Physician And Sportsmedicine	2.30	Orthopedics; Primary Health Care; Sport Sciences	SCIE & SSCI	3.09	6	23	2.29
19	Primary Care Diabetes	2.90	Endocrinology & Metabolism; Primary Health Care	SCIE & SSCI	5.00	7	23	2.29
20	Family Medicine And Community Health	6.10	Primary Health Care	ESCI	3.50	4	10	0.99

ACPA: Average citation per articles, N: Document Count, JIF: Journal impact factor for 2022 years, HI: H-index.

Appendix 4. Top 15 Most Cited Studies on Hypertension in the PHC Research Area

Rank	Title	Journal	JIF	Authors	Year	C
1	Randomized controlled trials: Do they have external validity for patients with multiple comorbidities?	Annals of Family Medicine	4.4	Fortin, M; Dionne, J; (...); Lapointe, L	2006	217
2	Prehypertension and cardiovascular morbidity	Annals of Family Medicine	4.4	Liszka, HA; Mainous, AG; (...); Egan, BM	2005	168
3	Why do GPs not implement evidence-based guidelines?A descriptive study	Family Practice	2.2	Cranney, M; Warren, E; (...); Walley, T	2001	149
4	Do whole-grain oat cereals reduce the need for antihypertensive medications and improve blood pressure control?	Journal of Family Practice	0.6	Pins, JJ; Geleva, D; (...); Cherney, LM	2002	119
5	Why hypertensive patients do not comply with the treatment	Family Practice	2.2	Gascón, JJ; Sánchez-Ortuño, M; (...); Saturno, PJ	2004	102
6	Race rural residence, and control of diabetes and hypertension	Annals of Family Medicine	4.4	Mainous, AG; King, DE; (...); Pearson, WS	2004	101
7	Cultural factors and patients' adherence to lifestyle measures	British Journal of General Practice	5.9	Serour, M; Alqhenaei, H; (...); Ben-Nakhi, A	2007	100
8	Integration of Depression and Hypertension Treatment: A Pilot, Randomized Controlled Trial	Annals of Family Medicine	4.4	Bogner, HR and de Vries, HF	2008	96
9	Health Coaching by Medical Assistants to Improve Control of Diabetes, Hypertension, and Hyperlipidemia in Low-Income Patients: A Randomized Controlled Trial	Annals of Family Medicine	4.4	Willard-Grace, R; Chen, EH; (...); Thom, DH	2015	95
10	Hypertension guideline recommendations in general practice: awareness, agreement, adoption, and adherence	British Journal of General Practice	5.9	Heneghan, C; Perera, R; (...); Glasziou, P	2007	95
11	Diagnosis of Secondary Hypertension: An Age-Based Approach	American Family Physician	4.0	Viera, AJ and Neutze, DM	2010	94
12	Educational and organisational interventions used to improve the management of hypertension in primary care: a systematic review	British Journal of General Practice	5.9	Fahey, T; Schroeder, K and Ebrahim, S	2005	84
13	The concept and definition of therapeutic inertia in hypertension in primary care: a qualitative systematic review	BMC Family Practice	2.9	Lebeau, JP; Cadwallader, JS; (...); Vermeire, E	2014	82
14	Self-monitoring and other non-pharmacological interventions to improve the management of hypertension in primary care: a systematic review	British Journal of General Practice	5.9	Glynn, LG; Murphy, AW; (...); Fahey, T	2010	81
15	Quality of clinical primary care and targeted incentive payments: an observational study	British Journal of General Practice	5.9	Steel, N; Maisey, S; (...); Howe, A	2007	81

JIF: Journal impact factor for 2022 years, C: Citation

Appendix 5. Hyper Tension Articles' LDA Topic Modeling Findings as a Word Cloud for Abstracts and Titles

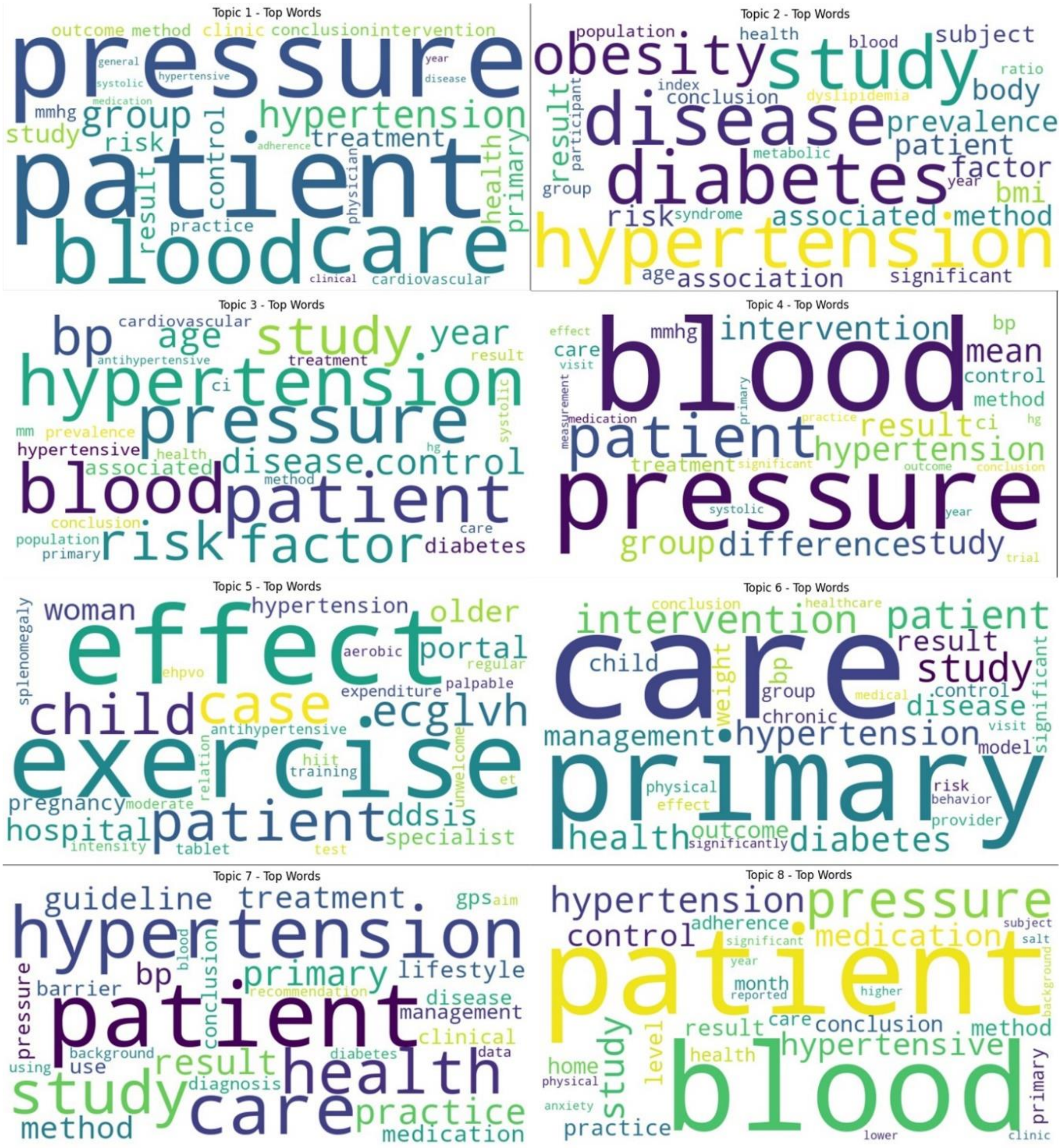

Supplement: Supplementary file 1 [file medi-103-e40482-s001.pdf]
